# Supplementary material for: Role of m6A RNA Methylation in Thyroid Cancer Cell Lines
Source: Int J Mol Sci. 2022 Sep 29;23(19):11516. doi: 10.3390/ijms231911516 (PMC9569446; doi:10.3390/ijms231911516)
Supplement: Supplementary file 1 [file ijms-23-11516-s001.zip › Supplementary material.pdf]

Supplementary material

Supplementary Table

Table S1 : Primer sequences used in RT-qPCR

Oligo name	Sequence
ACTB Forward	TTGTTACAGGAAGTCCCTTGCC
ACTB Reverse	ATGCTATCACCTCCCCTGTGTG
METTL3 Forward	ACCATTCCAAGCTCTCTGCC
METTL3 Reverse	AGTCGAGTCCTGTTCTGCAC
DDI2 Forward	GGATGGCGTGTCACGTAGAT
DDI2 Reverse	CCTGGCTGACCTGTTGTTCT

Supplementary Figure

Figure S1: Number of 8505C or SW1736 viable cells after treatments with negative control (nc) or with METTL3 specific siRNAs (siRNA 1 and siRNA 2). After staining with trypan blue, only viable cells were considered. $n = 4$. *** $p < 0.001$.
