# Supplementary figures and images for: Role of m6A RNA Methylation in Thyroid Cancer Cell Lines
Source: Int J Mol Sci. 2022 Sep 29;23(19):11516. doi: 10.3390/ijms231911516 (PMC9569446; doi:10.3390/ijms231911516)

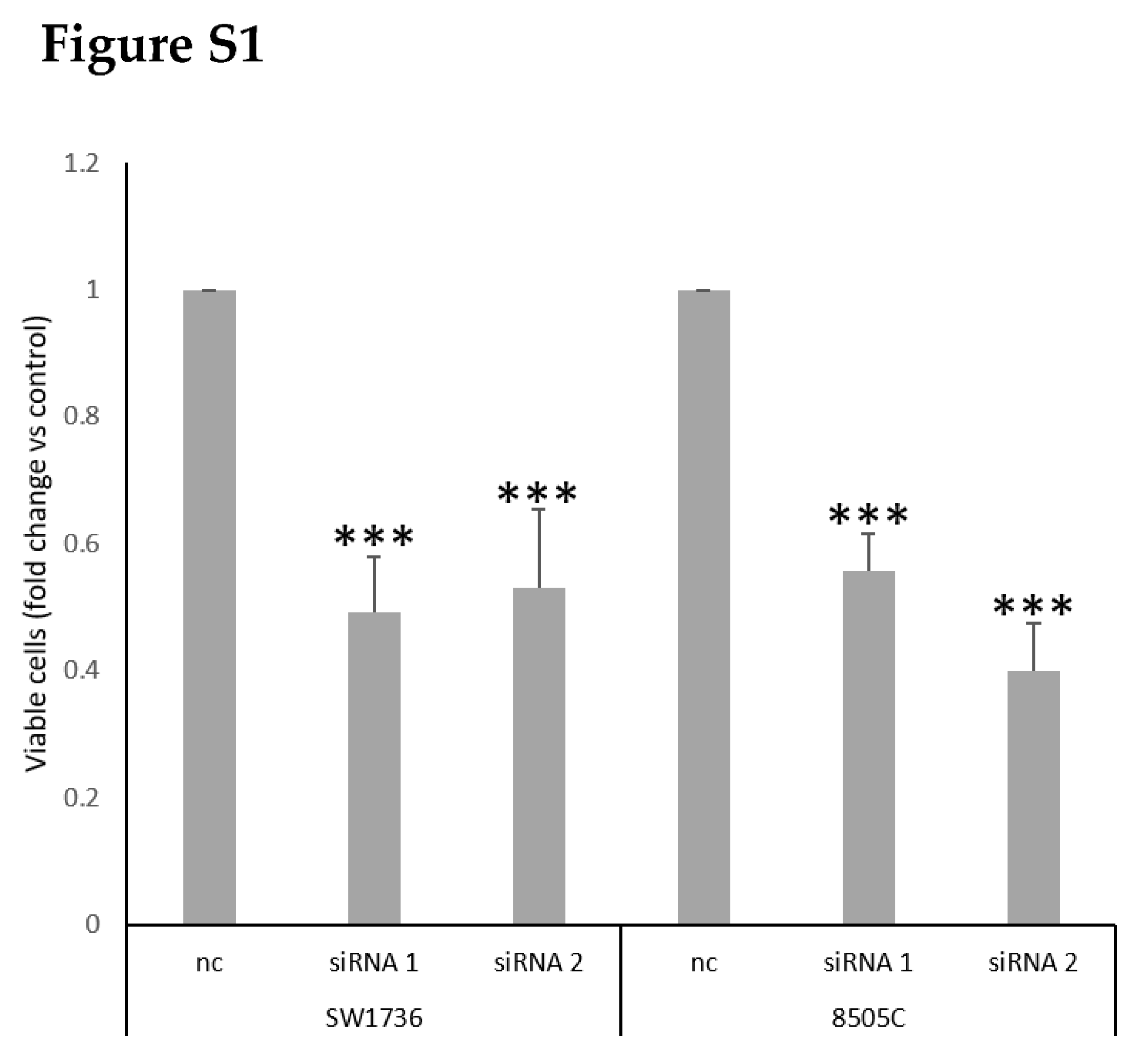

Supplement: Supplementary file 1 [file ijms-23-11516-s001.zip › Supplementary Figure S1.tif]
